# Toxicological Effects of Sulphate of Quinine

**Published:** 1844-02

**Authors:** 


*Toxicological Effects of Sulphate of Quinine.*—It has become a prevalent practice in the Southwestern States to employ sulphate of quinine in large doses. We have administered it in doses of 15 grains, at intervals of two hours, until, in one case, the patient took during the day 90 grains. The disease was a malignant intermittent, generally termed congestive fever, and the remedy had the desired effect of preventing the return of the chill, and establishing convalescence. Cases are reported in which much larger doses were given. In a few instances as much as a drachm has been taken at one time. But this practice, it appears, is not without danger.

"M. Melier lately read a memoir to the Academy of Sciences, in which he gave an account of some experiments of Magendie on the effects of poisonous doses of quinine. Dogs were poisoned with this substance, and on dissection distinct fluidity of the blood was observed, and morbid enlargement of the parenchyma of the lungs. M. Melier therefore cautions against the practice of administering the large doses."—*Edinburgh Med. and Surg. Jour.*

